# Supplementary material for: Expression of Lumican and Osteopontin in Perivascular Areas of the Glioblastoma Peritumoral Niche and Its Value for Prognosis
Source: Int J Mol Sci. 2024 Dec 29;26(1):192. doi: 10.3390/ijms26010192 (PMC11720198; doi:10.3390/ijms26010192)
Supplement: Supplementary file 1 [file ijms-26-00192-s001.zip › ijms-3341772-supplementary.pdf]

Expression of Lumican and Osteopontin in Perivascular Areas of the Glioblastoma Peritumoral Niche and its Value for Prognosis

Salinas, M.D.[1,2]; Rodriguez, P.[1,2]; Rubio, G.[1]; Valdor, R.[1,2*]

[1] *Biochemistry, Molecular Biology B, and Immunology Department, University of Murcia (UMU), 30120, Murcia, Spain.*

[2] *Unit of Autophagy, Immune response and Tolerance in Pathologic Processes, Biomedical Research Institute of Murcia (IMIB), 30120, Murcia, Spain.*

***Correspondence:** Rut Valdor, rut.valdor@um.es, ORCID: 0000-0002-2681-0779

Supplementary material and methods

Immunohistochemistry, immunofluorescence, and microscopy

After surgical resection, tissue specimens were fixed in 4% buffered formaldehyde (Panreac Quimica), embedded in paraffin, processed by the Pathology facility (IMIB). Three-micrometer thick serial sections were obtained from paraffin-embedded samples using an automatic rotary microtome (Thermo Scientific). The sections were then incubated overnight at 4ºC with specific primary antibody goat anti-GFAP (Ready-to-Use, IR52461-2, Dako Agilent). Sections were then incubated with the respective anti-goat secondary HRP labeled polymer system (Vector ImmPress). The immunoreaction was revealed by using a 3-´Diaminobencidine 3,3’-diaminobenzidine (DAB) substrate kit (Dako DAB substrate kit) which identifies positive immunoreaction as a dark brown (DAB) precipitate. Finally, sections were counterstained with Mayer’s hematoxylin (LLG06272066, Carlo Erba Reagents). For hematoxylin-eosin (HE) staining, sections were incubated with Mayer’s hematoxylin (LLG06272066, Carlo Erba Reagents) and Shandon Eosin-Y (6766009, Thermofisher Scientific) The stained markers were visualized using the Slide Viewer software (3DHISTECH).

Images captured the peritumoral area, with a focus on blood vessels for perivascular analysis, all displaying a 100-micron visual scale. HE staining was used for histology and tumor infiltration assessment. Further analysis involved evaluating GFAP staining for microglia presence and morphology, categorizing tissue areas in peritumoral and invasion front.

Supplementary tables and figures

Table S1. Patient information. ♀ female, ♂ male, ATRX expression of nuclear ATRX, S = Surgery, R = Radiotherapy, C = Chemotherapy, RC = Radio-Chemotherapy.

Patient / Gender	Age at diagnosis	WHO grade	ATRX - Expression	P53 - Expression	Treatment	CMA activity
GLIO-12♂	64,5	IV	Retained	Positive	S + RC	Low
GLIO-19♂	66,7	IV	Retained	Positive	S + RC	Low
GLIO-20♀	52,9	IV	Lost	Positive	S + C	Medium
GLIO-22♂	40,3	IV	Retained	Positive	S + RC	Medium
GLIO-23♀	65,4	IV	Retained	Positive	S + RC	High
GLIO-24♀	87,3	IV	Retained	Positive	S + RC	Medium
GLIO-27♂	79,3	IV	Lost	Negative	S	Medium
GLIO-30♂	32,7	IV	Retained	Positive	S + RC	High
GLIO-31♂	65,0	IV	Retained	Positive	S + RC	Medium
GLIO-32♀	61,2	IV	Retained	Positive	S + RC	Low
GLIO-34♀	54,9	IV	Retained	Positive	S + RC	Medium
GLIO-35♂	42,2	IV	Retained	Negative	S + RC	Medium
GLIO-36♀	71,7	IV	Retained	Positive	S + RC	Low
GLIO-37♂	50,3	IV	Retained	Positive	S + RC	Medium
GLIO-38♂	61,3	IV	Retained	Negative	S + RC	High
GLIO-41♀	54,8	IV	Retained	Negative	S + RC	High
GLIO-42♂	51,3	IV	Retained	Negative	S + RC	Medium
GLIO-44♂	50,2	IV	Retained	Negative	S + RC	Low
GLIO-46♂	61,8	IV	Retained	Positive	S + C	Low
GLIO-47♂	48,5	IV	Lost	Positive	S + C	High
GLIO-48♂	51,0	IV	Retained	Negative	S + RC	High
GLIO-51♀	51,0	IV	Lost	Negative	S + RC	Medium
GLIO-52♂	32,7	IV	Retained	Positive	S + RC	Low
GLIO-53♀	47,7	IV	Retained	Negative	S + RC	High
GLIO-54♀	59,4	IV	Retained	Positive	S	Medium
GLIO-55♀	65,0	IV	Lost	Negative	S	Low
GLIO-56♀	52,4	IV	Lost	Positive	S + RC	High
GLIO-57♀	59,5	IV	Retained	Negative	S + RC	High
GLIO-59♂	69,1	IV	Retained	Positive	S + RC	Medium
GLIO-60♂	54,5	IV	Retained	Negative	S + RC	Medium
GLIO-62♀	61,2	IV	Retained	Positive	S + RC	Medium
GLIO-63♂	53,0	IV	Retained	Positive	S + RC	Low

GLIO-65♀	66,0	IV	Retained	Positive	S + RC	Low
GLIO-66♂	64,2	IV	Retained	Positive	S + RC	Low

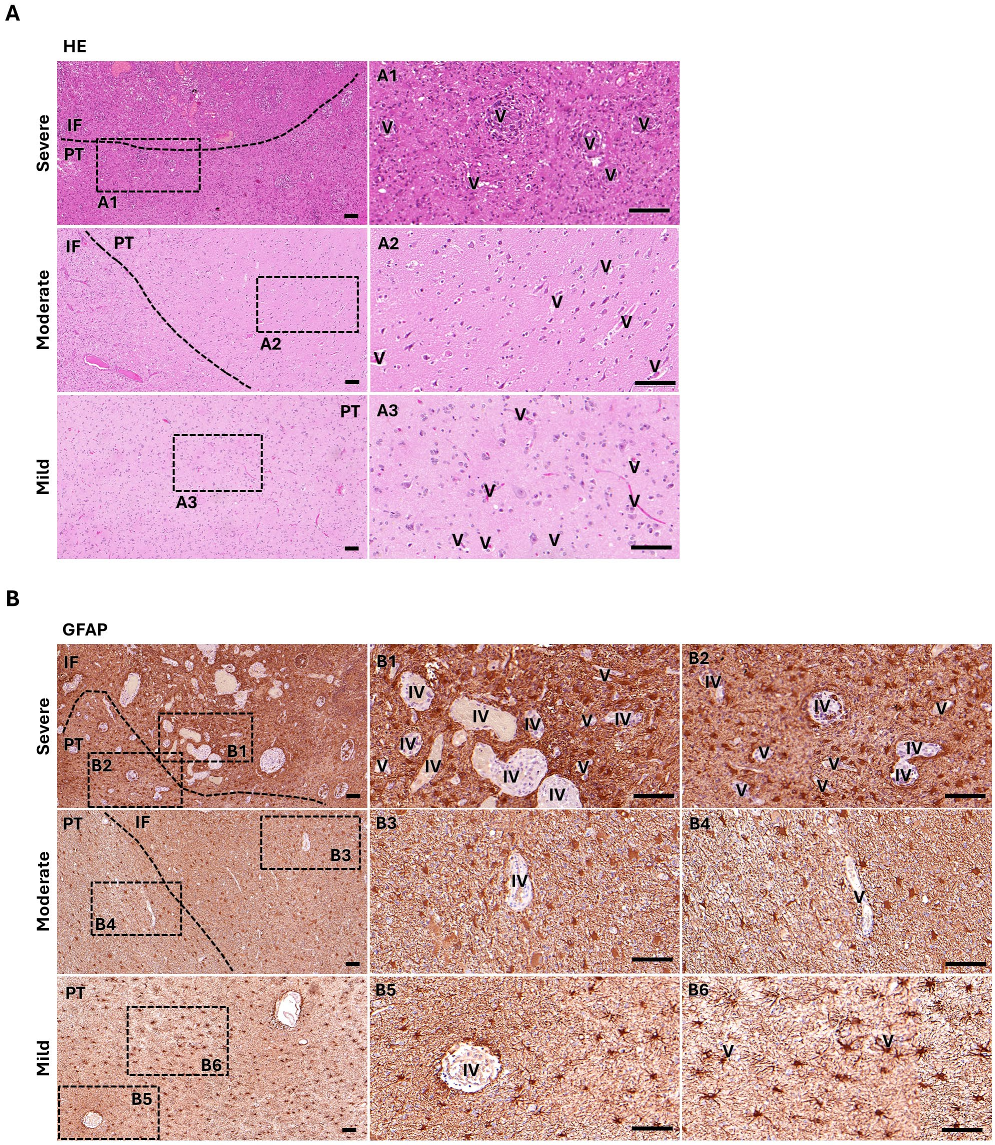

Fig. S1. Histology evaluation and peritumoral areas identification in glioblastoma tissue samples. (**A**) Hematoxylin and Eosin (HE) staining in the peritumoral areas (PT) of the invasion front (IF) of GB cancer classified from severe to mild according to the level of GB invasion. Dashed line marks the separation between the PT zones and the beginning of the IF. A1 to A3 magnifications show perivascular areas with microvessels (v). Scale: 100 µm. (**B**) Staining of GFAP, as specific marker of astrocytes that identifies GB cells of glial origin, in peritumoral areas (PT) of the invasion front (IF). Dashed line marks the separation between the PT zones and the beginning of the IF. Center and right panels show magnification of IF (B1 and B3) and PT (B2, B4 and B6) areas. In mild patients (B5), only infiltrated blood vessels (IV) were found. Scale bars: 100 µm.

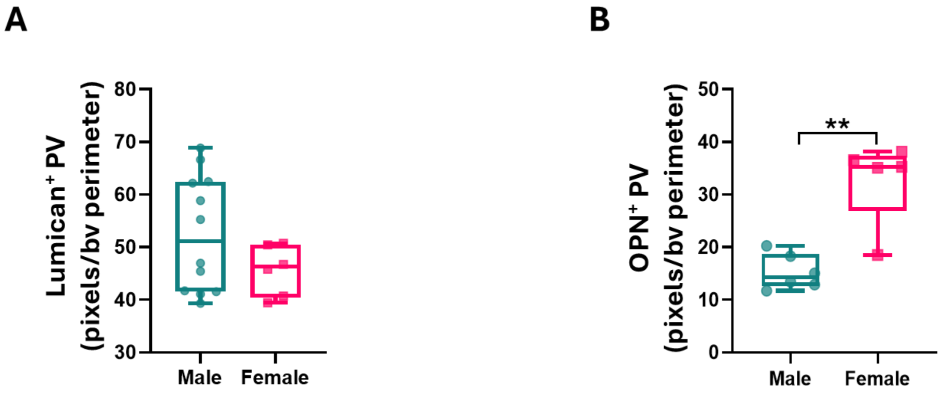

Figure S2. Lumican (**A**) and OPN (**B**) perivascular quantification grouped by gender in the of the quantification of positive particles in the perivascular (PV) microenvironment, age range 30-65 years in the cohort of mild-to-moderate GB patients. Boxplots diagrams expressed as positive pixels per blood vessel (bv) perimeter. Quantifications were performed in at least four fields and in a minimum of 5 blood vessels; **$p<0.001$.
